# Myeloperoxidase (MPO) Enzymatic Activity, but Not Its Protein Concentration, Is Associated with the Risk of Type 2 Diabetes in Females, Regardless of Obesity Status

**DOI:** 10.3390/antiox15010130

**Published:** 2026-01-19

**Authors:** Alessandro Trentini, Raffaella Riccetti, Domenico Sergi, Juana Maria Sanz, Riccardo Spaggiari, Valentina Rosta, Gianmarco Mola, Angelina Passaro, Carlo Cervellati

**Affiliations:** 1 Department of Environmental and Prevention Sciences, University of Ferrara, 44121 Ferrara, Italy; alessandro.trentini@unife.it (A.T.); raffaella.riccietti@unife.it (R.R.); 2 Department of Translational Medicine and for Romagna, University of Ferrara, 44121 Ferrara, Italy; domenico.sergi@unife.it (D.S.); riccardo.spaggiari@edu.unife.it (R.S.); valentina.rosta@unife.it (V.R.); gianmarco.mola@unife.it (G.M.); angelina.passaro@unife.it (A.P.); carlo.cervellati@unife.it (C.C.)

**Keywords:** neutrophil-derived myeloperoxidase, myeloperoxidase enzymatic activity, type 2 diabetes mellitus, obesity, females

## Abstract

To date, neutrophil-derived myeloperoxidase (MPO), a key mediator of inflammation and oxidative stress, has predominantly been assessed in peripheral fluids by protein concentration rather than enzymatic activity, mainly due to methodological limitations. However, MPO activity directly reflects the enzyme’s cytotoxic potential and pathogenic role in inflammatory diseases. To address this gap, we employed an optimized immunocapture assay to evaluate MPO activity, specific activity, and protein concentration in females with type 2 diabetes mellitus (T2DM), a condition tightly linked to chronic low-grade inflammation and obesity. Our findings revealed that females with T2DM exhibited nearly three-fold higher serum MPO activity and more than two-fold greater specific activity compared to controls with no differences in MPO protein concentration. Notably, MPO-specific activity remained significantly associated with T2DM (*p* < 0.01 to *p* < 0.001 across multivariate models), even after adjusting for age and dual-energy X-ray absorptiometry-derived measures of total and regional fat mass. Only android/gynoid fat distribution retained marginal significance in these models. This study is the first demonstration that MPO enzymatic activity, rather than protein concentration, is independently linked to T2DM in females. These findings underscore the importance of assessing functional MPO activity in the context of metabolic disease and support its potential role as a pathophysiological marker.

## 1. Introduction

Obesity, largely characterized by the accumulation of visceral adipose tissue, is associated with increased infiltration of immune cells within adipose depots, predominantly macrophages. These cells play a pivotal role in promoting the chronic low-grade inflammatory state and oxidative stress typical of obesity, which in turn drive insulin resistance, a key pathogenetic factor in the development of obesity-related metabolic comorbidities, including type 2 diabetes mellitus (T2DM) and metabolic syndrome [1,2,3]. Abnormalities related to both overall obesity, commonly assessed by body mass index (BMI), and central obesity substantially increase cardiovascular disease (CVD) risk in women, particularly after menopause [4,5,6]. This increased risk is largely attributable to enhanced fat deposition, especially within the visceral compartment, a hallmark of the menopausal transition [7].

Myeloperoxidase (MPO), a member of the heme-peroxidase enzyme family, is considered a major contributor to the pro-inflammatory and pro-oxidant actions of neutrophils and macrophages [8,9,10]. This enzyme plays a crucial role in innate immune system defense, with chronic inflammation being a potential trigger of its expression and release from neutrophils and macrophages [10]. Upon activation, MPO-expressing cells release the protein primarily into the phagolysosomal compartment, with a smaller fraction being secreted extracellularly [10]. MPO also facilitates NADPH oxidase-2 (NOX2) assembly, promoting the generation of superoxide radical anions (O_2_^−^•), which are rapidly converted to hydrogen peroxide (H_2_O_2_) [10]. MPO converts H_2_O_2_ and halides, such as Cl^−^ and Br^−^, into hypohalous acids, hypochlorous acid (HOCl), and hypobromous acid (HOBr), respectively, which are potent antimicrobial agents [10,11,12]. However, HOCl lacks microbial specificity, and substantial evidence indicates that it causes significant oxidative damage to host neutrophil constituents, as well as the endothelium and other tissues [10]. These collateral effects mainly occur as a consequence of excessive or inappropriate activation of leukocytes, along with chronic low-grade inflammation [8].

Elevated MPO levels have diagnostic and prognostic value for various cardiovascular diseases, being able to predict poor outcomes, and serving as a valuable biomarker for cardiovascular risk stratification [13,14]. In keeping with this, MPO has been linked with a plethora of diseases characterized by an inflammatory background as well as vascular oxidative stress [13,14,15,16,17,18,19]. Nevertheless, whether MPO levels are upregulated in diseases characterized by inflammatory responses, including type 2 diabetes (T2DM), remains a matter of debate, due to some contrasting findings [20,21,22]. Indeed, despite low-grade chronic inflammation being at the interphase between obesity, insulin resistance, and T2DM, some, but not all, animal and human studies identified a clear connection between MPO serum levels and insulin resistance or T2DM [23,24,25,26].

In our view, the inconsistency in findings regarding MPO in T2DM may partly stem from the fact that these findings have been solely based on measuring protein concentration, rather than its activity. Indeed, not all MPO is present in an inactive or partially active form within granulocytes and the extracellular environment [27,28]. Notably, the inactive form appears to have immunomodulatory properties that are independent from MPO activity [27,28]. Conversely, the active form of the enzyme is responsible for non-specific biological damage, which can significantly amplify the inflammatory state and oxidative stress [29,30]. In line with this, we found that enzymatic activity, particularly specific activity (measured as the ratio between activity and mass), is more effective in identifying patients with diseases characterized by elevated inflammation [31]. Functional biomarkers reflecting active pathophysiological processes, such as MPO, have gained increasing interest, as they integrate inflammatory and oxidative stress pathways directly involved in disease progression. In contrast, traditional markers, such as genetic predisposition and static clinical measures, are limited in their ability to capture the dynamic biology of cardiometabolic disease, reducing their predictive value for complications in T2DM [32,33].

Building on these premises, this study sought to examine the association between MPO (measured as protein concentration, activity, and specific activity) and T2DM in females, and to explore whether body fat distribution influences this relationship.

## 2. Methods

### 2.1. Study Cohort

The study included a total of 194 females, of whom 141 were of healthy and 53 had T2DM. Participants were recruited at the Menopause and Osteoporosis Centre, University of Ferrara (Italy). This study was designed and conducted in accordance with the Code of Ethics of the World Medical Association (Declaration of Helsinki) and was approved by the ethics committee of the University-Hospital of Ferrara (207/2019/Sper/UniFe, approved on 20 May 2019). Written informed consent was obtained from each participant before inclusion into the study. Inclusion criteria comprised females of reproductive age or postmenopausal status, the latter defined as at least 12 consecutive months of amenorrhea, in accordance with the recent ReSTAGE modification of the Stages of Reproductive Aging Workshop (STRAW) staging criteria [34].

Exclusion criteria were the presence of chronic diseases other than T2DM (thrombo-embolism, neurodegenerative diseases, significant systemic infections, autoimmune diseases, malignant neoplastic diseases, or renal failure); menopause hormonal therapy (estrogen or estrogen–progestin) in the 6 months prior to the enrollment into the study; frequent use (on average, more than twice a week) of vitamin supplements or medications (anti-inflammatory, analgesic, anti-allergic, and antidepressant); history of cardiovascular diseases; habitual consumption of alcoholic beverages (>30 g of alcohol/day); or a current smoking habit.

The diagnosis of T2DM was made by following the American Diabetes Association (ADA) criteria.

### 2.2. Biochemical Analysis

Fasting blood samples were processed by centrifugation at 1600× *g* for 15 min to separate serum or plasma, which was then aliquoted and stored at −80 °C until further analysis.

Glucose, insulin, total cholesterol, HDL cholesterol, triglycerides, apolipoprotein A-1 (ApoA), and apolipoprotein B100 (ApoB) were assayed using standard enzymatic colorimetric techniques (Beckman Coulter, Brea, CA, USA) at the Central laboratory of S. Anna University Hospital, Ferrara; LDL cholesterol levels were calculated using Friedewald’s formula.

HOMA-IR was used to estimate insulin resistance by using the following formula:

$$HOMA-IR=\frac{fasting\;serum\;insulin\left(\frac{\mathrm{\mu U}}{\mathrm{ml}}\right)\times fasting\;plasma\;glucose(\frac{\mathrm{mmol}}{L})}{22.5}$$

Glucose concentrations were initially expressed in mg/dL and were converted to mmol/L, as required for the HOMA-IR formula, using a conversion factor of 0.0555.

### 2.3. Assay of MPO Activity

The measurement of MPO enzymatic activity was performed as previously described [31]. Briefly, ELISA microplates (Nunc-Maxisorp, Thermo Scientific, Cat. No. 446612, Milan, Italy) were coated with 100 μL of anti-MPO polyclonal antibodies (Calbiochem (Merck Life Science, Milan, Italy), Cat. No. 475915) diluted 1:500 in 0.2 M sodium bicarbonate buffer (pH 9.4) and incubated overnight at 4 °C. After incubation, wells were washed three times with 300 μL of wash buffer (0.15 M NaCl, 0.1 M NaH_2_PO_4_, pH 7.2, and 0.05% Tween-20), followed by blocking with 300 μL of 5% BSA in wash buffer for 1 h at room temperature with gentle agitation.

Following three additional washes, 100 μL of serum (diluted 1:8) or MPO standard (0.39–25 ng/mL; Calbiochem, Cat. No. 475911) prepared in 1% BSA in wash buffer (without Tween-20) were added in duplicate. After 1 h of incubation at room temperature with gentle agitation, wells were washed four times and incubated with 50 μL of 392 μM H_2_O_2_ and 50 μL of 200 μM AmpliFlu Red (Sigma-Aldrich (Milan, Italy), Cat. No. 90101), both diluted in 20 mM citrate buffer (pH 6) containing 80 mM NaBr. Final assay concentrations were 196 μM H_2_O_2_ and 100 μM AmpliFlu Red.

Fluorescence of the reaction product (resorufin) was measured every 30 s for 10 min at 37 °C using a microplate fluorimeter (Tecan Infinite M200pro, Männedorf, Switzerland) with excitation/emission wavelengths of 535/590 nm. Enzymatic activity (U/mL) was calculated from a standard curve generated with known concentrations of resorufin, as previously detailed [31].

The assay showed an intra- and inter-assay coefficient of variation of 5.8% and 10.4%, respectively, with a limit of detection (LoD) of 0.074 μU.

### 2.4. Assessment of MPO Concentration

The concentration of total MPO was determined by a commercially available ELISA kit by following the manufacturer’s instructions with some changes (Human MPO, Aviscera (Guidonia Montecelio, Italy), Cat. No. SK00172-09). Briefly, the capture antibody provided in the kit was diluted 1:100 with coating buffer (100 mM sodium bicarbonate, pH 9.0), and 100 mL was dispensed into the wells of an ELISA microplate (Nunc-Maxisorp, Thermo Scientific, Cat. No. 446612). After an overnight incubation at 4 °C, the wells were washed three times with 300 mL/well of wash buffer (WB, 0.05% *v*/*v* Tween-20 in PBS, pH 7.4), followed by the addition of 300 mL/well of Blocking Buffer (5% BSA in WB). After 1 h of incubation at room temperature and three washing steps with 300 mL/well of WB, 100 mL of MPO standard (dilution range 1.56–400 ng/mL), serum samples (diluted 1:8 with dilution buffer), or dilution buffer (1% BSA in PBS, pH 7.4), as the blank, were dispensed in duplicate in the designated wells of the plate. After 1.5 h of incubation with slow agitation at room temperature, the plate was washed four times with 300 mL/well of WB and 100 mL/well of detection antibody (diluted 1:100 in 1% BSA in PBS, pH 7.4, and 0.1% *v*/*v* Tween-20) was dispensed in the well of the microplate and incubated for 1 h with slow agitation at room temperature. At the end of the incubation, the plate was washed four times with 300 mL/well of WB and 100 mL/well of substrate solution (4 mM 3,3′,5,5′-tetramethylbenzidine, 2 mM H_2_O_2_, and in 20 mM citrate buffer at pH 5.0) was added to each well, and incubated for exactly 5 min at room temperature in the dark with slow agitation. The reaction was stopped by adding 100 mL/well of Stop Solution (2M H_2_SO_4_), and the absorbance was read in a microplate reader (Tecan Infinite M200pro) at 450 nm, with a reference wavelength set at 540 nm. The amount of MPO was then calculated by interpolation with the standard curve.

### 2.5. Assessment of MPO-Specific Activity

MPO-specific activity is the measure of its enzymatic activity normalized to the amount of its protein. It was calcualted by dividing MPO acitivity by MPO concentration, and expressed is in U/µg.

### 2.6. Assessment of Anthropometric Indexes of Adiposity and Body Composition Using DXA

Body mass, height, and body mass index (BMI) were measured according to standard protocols by trained personnel. Obesity was defined using the conventional cut-off of body mass index (BMI) ≥ 25 kg/m^2^. Body composition was measured by DXA (Hologic Discovery; software version APEX 3.3.0.1, Bedford, MA, USA). The instrument software (version 1.5) provides estimates of lean tissue mass, fat mass (FM), and bone mineral mass for the total body and for standard body regions. Using specific anatomic landmarks, regions for the head, arms, trunk (subdivided into two sub-regions: thorax and abdomen), and legs were distinguished, as reported elsewhere [35]. Total fat percentage was calculated as 100 × total FM/(total bone mineral content + total lean mass + total FM). Regional FM was calculated as 100 × regional FM/total FM. Moreover, the technology allows the estimation of other fat parameters, more specifically, visceral fat (the software can measure this type of adiposity with good analytical accuracy) and android/gynoid ratio.

### 2.7. Statistical Analysis

Continuous variables are presented as mean ± standard deviation (SD) and median (interquartile range). The Shapiro–Wilk test was used to assess normality. As all three MPO measures (protein concentration, activity, and specific activity) were non-normally distributed, group comparisons (two or three groups) were performed using the Mann–Whitney U test or Kruskal–Wallis test, respectively. These tests were used to verify possible association between these markers and TD2M, but also obesity, hypertension, and menopausal status.

A receiver operating characteristic (ROC) curve was performed to evaluate the diagnostic accuracy of the MPO markers in discriminating controls from diseased patients.

To satisfy the assumptions of multivariate analyses (primarily, multiple regression), MPO markers were log10-transformed. Subsequent evaluation of distributions and residuals confirmed that only MPO-specific activity fully met the criteria for normality and linearity. Consequently, associations involving this marker (log-transformed values) were assessed using Pearson’s correlation, while Spearman’s rank correlation was employed for the other two MPO markers.

Stepwise multiple regression analysis was then conducted with log-transformed MPO-specific activity as the outcome variable to verify the independence of associations identified in univariate analysis. Multicollinearity was assessed using the variance inflation factor (VIF), with values > 2.5 indicating exclusion of the affected variable. Variables were entered into the model if *p* ≤ 0.05 and removed if *p* > 0.10. A two-tailed *p*-value < 0.05 was considered statistically significant.

Based on the established statistical criteria, three multiple regression models were constructed. Each model incorporated the following: (1) variables significantly associated with MPO-specific activity in univariate analysis and (2) the clinically relevant covariates of age, menopausal status, and diabetes mellitus. Data analysis was performed using SPSS Statistics for Windows, version 26.0 (SPSS, Inc., Chicago, IL, USA), and a *p* < 0.05 was considered as statistically significant.

## 3. Results

### 3.1. Main Characteristics of the Study Population

The general characteristics of the study’s participants are summarized in Table 1. Compared to controls, females with T2DM were older (56 vs. 52 years, *p* < 0.01), more frequently postmenopausal (74% vs. 49%, *p* < 0.01), and had a higher prevalence of obesity and hypertension (both *p* < 0.001).

As expected, the T2DM group showed a higher BMI and a greater percentage of hypertension compared to controls (*p* < 0.001 for all).

A similar pattern was observed for clinical chemistry markers: HOMA-IR index, triglycerides, ApoA, HDL-C, and glucose levels were significantly higher in T2DM than in controls, with *p*-values ranging from <0.01 to <0.001. In contrast, total cholesterol and LDL-C levels were slightly but significantly higher in controls compared with the T2DM group (*p* < 0.05).

As expected, DXA-derived fat mass (FM) parameters tended to be higher in controls than in individuals with T2DM, with highly significant differences (*p* < 0.001) observed for visceral fat-related indices (Supplementary Table S1).

### 3.2. MPO Activity, Concentration, and -Specific Activity in T2DM

**Figure 1:**
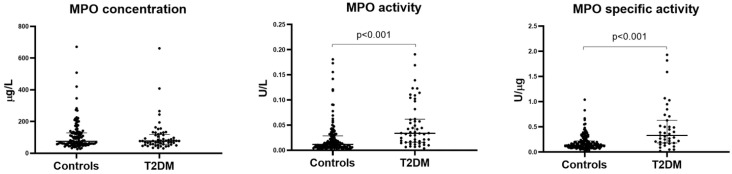
Levels of MPO activity, MPO concentration, and MPO-specific activity in controls and T2DM patients. Both MPO activity and MPO-specific activity were significantly higher in T2DM patients compared to controls. On the contrary, MPO concentration did not change between the two groups.

As illustrated in Figure 1, the median MPO activity was nearly three times higher in individuals with T2DM than in the controls (0.0338 U/L, interquartile range—IR: 0.0160–0.0619 vs. 0.0116 U/L, IR: 0.0063–0.0286; *p* < 0.001). In contrast, MPO concentrations showed minimal variation (77.5 ng/mL, IR: 53.2–115.5 vs. 76.7 ng/mL, IR: 59.4–129.4). This combination of results led to significantly increased MPO-specific activity in T2DM compared to the controls (0.334 U/µg, IR: 0.182–0.62 vs. 0.141 U/µg, IR: 0.98–0.221).

The ability of MPO activity and MPO-specific activity to discriminate between controls and individuals with T2DM was further evaluated using receiver operating characteristic (ROC) curve analysis. Both markers demonstrated moderate discriminatory performance, with areas under the curve (AUCs) of 0.747 for MPO activity and 0.757 for MPO-specific activity (Supplementary Figure S1). The optimal balance between sensitivity and specificity was determined by selecting the point closest to the upper-left corner of the ROC curve. For MPO activity, a cut-off value of 0.017 U/L provided the best compromise, yielding a sensitivity of 75% and a specificity of 65%. Similarly, for MPO-specific activity, an optimal cut-off of 0.174 U/µg was identified, corresponding to a sensitivity of 79% and a specificity of 65%.

**Figure 2:**
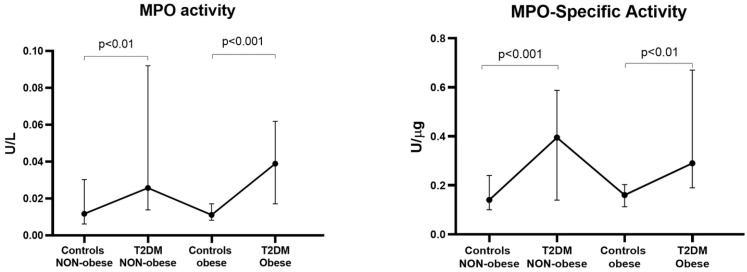
Influence of obesity on MPO activity and MPO-specific activity in controls and T2DM patients. Both MPO activity and MPO-specific activity were higher in T2DM patients than controls, regardless of obesity status.

Since obesity, a key risk factor for the development of T2DM, was the sole categorical variable showing significant associations with elevated MPO activity (+19%, *p* < 0.01 in obese compared to non-obese) and MPO-specific activity (+31%, *p* < 0.01), we investigated its potential influence on MPO levels (Figure 2). We analyzed four distinct subgroups: (1) controls (no T2DM) non-obese; (2) controls obese; (3) T2DM non-obese; and (4) T2DM obese. The analysis revealed that T2DM status, rather than obesity per se, served as the primary determinant of both MPO activity and MPO-specific activity. Notably, both markers were consistently elevated in T2DM subjects compared to controls, regardless of obesity status.

The same considerations can be made for other factors potentially influencing MPO: hypertension, menopausal status, and age, with only the last being significantly correlated with MPO concentration (r = −0.161, *p* < 0.05, ) but not with activity or MPO-specific activity.

### 3.3. Relationship Between Myeloperoxidase (MPO) (Activity, Concentration, and -Specific Activity), Metabolic Parameters, and Body Fat Distribution

Univariate correlation of the three MPO measures is displayed in Table 2. Regarding blood metabolic biomarkers, both MPO activity and MPO-specific activity showed inverse correlations with total cholesterol (*p* < 0.01 for both MPO measures), LDL cholesterol (*p* < 0.01 for both), APOA (*p* < 0.05 for both), and HDL cholesterol (*p* < 0.05 and *p* < 0.01, respectively). Finally, both MPO activity and MPO-specific activity strongly and positively correlated with both insulin and HOMA-IR.

BMI showed a stronger correlation with both MPO activity and MPO-specific activity (r = 0.305, *p* < 0.001 and r = 0.293, *p* < 0.001, respectively), compared to total body fat (r = 0.164, *p* < 0.05 and r = 0.224, *p* < 0.01, respectively). Overall, the association between DXA-derived measures of total and regional FM and MPO-specific activity were stronger than those between these FM parameters and MPO activity. In particular, MPO-specific activity exhibited highly significant correlations with all central body fat measures except trunk FM (*p* < 0.01), whereas MPO activity exhibited only modest associations. Notably, the only inverse correlation emerged by these analyses was that between MPO activity and MPO-specific activity with percentage of legs FM (*p* < 0.05 and *p* < 0.01, respectively).

**Figure 3:**
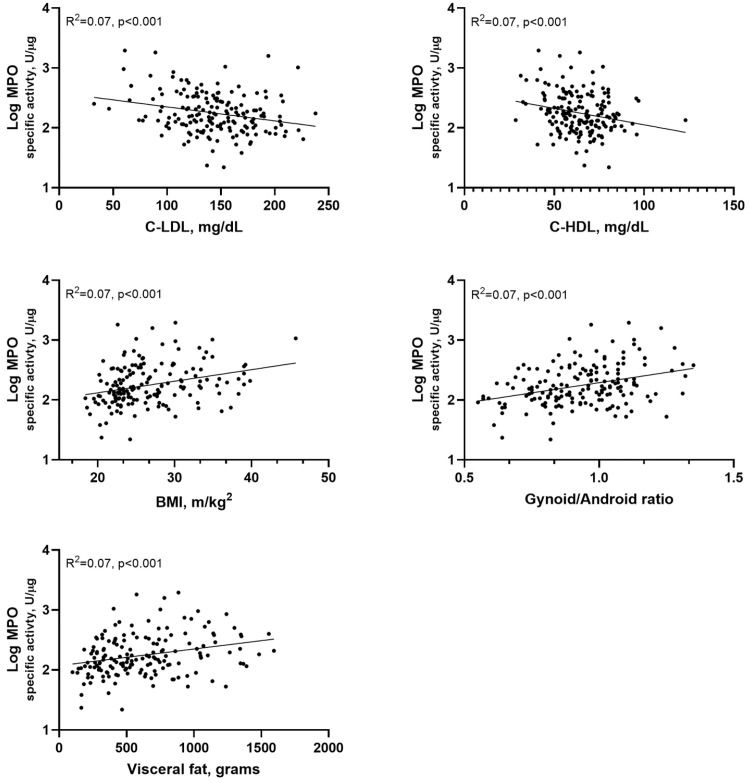
Correlation between Log-transformed MPO-specific activity and parameters of lipid profile and overall and central obesity. MPO-specific activity was inversely correlated (as assessed by Pearson’s correlation test) with C-LDL and C-HDL, and was positively correlated with BMI, gynoid/android ratio, and visceral fat.

The correlation analysis between Log-transformed MPO-specific activity and the parameters of the lipid profile and central obesity is graphically represented in Figure 3.

### 3.4. Variables, Including T2DM, Independently Associated with MPO-Specific Activity

Multivariate stepwise regression analysis was conducted to assess whether the association of MPO-specific activity with T2DM was influenced by potential confounders (Table 3). Due to high collinearity among certain variables (e.g., total FM, percentage of total FM, BMI, and measures of central adiposity), the analysis was performed using three separate regression models rather than a single combined model. Variables were primarily selected from those showing significance in univariate analysis.

The results revealed that diabetes (*p* < 0.01 in Model 1; *p* < 0.001 in Models 2 and 3) and LDL-C (*p* < 0.05 in all models) were the strongest independent correlates of MPO-specific activity. Among body adiposity measures, only the android/gynoid ratio (*p* < 0.05) emerged as an independent predictor of MPO-specific activity. Notably, substituting BMI with either absolute total FM or percentage FM in Model 1 yielded results consistent with those presented in Table 3.

## 4. Discussion

Chronic inflammation and oxidative stress are widely recognized as key contributors to the onset and progression of T2DM and its cardiovascular complications [36].

Myeloperoxidase (MPO), along with NADPH oxidase (NOX), is one of the few enzymes in phagocytes capable of converting pro-inflammatory signals into reactive species [11,12,37]. This unique function places MPO as a potentially critical mediator in diseases linked to chronic or acute inflammation.

Despite this, MPO’s biologically plausible role in T2DM has remained controversial, due to conflicting data from published studies [20,21,22,23,24,25,26].

We propose that these discrepancies may arise from a methodological gap. Crucially, most studies in this field measure only MPO concentration rather than its enzymatic activity, which is a critical distinction since activity reflects the functional contribution of MPO to disease processes [27,28,38]. This gap is particularly relevant for MPO, as individuals may exhibit normal protein levels but impaired activity due to genetic variants, oxidative inactivation, pharmacological inhibition, or other, in part still unknown, endogenous modulators [30,39,40]. Furthermore, the limited studies evaluating MPO activity have relied on suboptimal techniques (e.g., direct spectrophotometric assay) that suffer from poor analytical specificity [41]. To address these shortcomings, we developed an immunocapture-based activity assay that combines anti-MPO antibody specificity with precise enzymatic quantification, enabling direct measurement of activity per unit of protein [19,31].

Based on these premises, we aimed to investigate whether MPO, measured as protein concentration, enzymatic activity, and specific activity, was associated with T2DM in females. Our most striking finding was that, although the concentration of MPO in the bloodstream did not differ between females with and without T2DM, the enzyme was significantly more active in those with the disease. This heightened activity, in the absence of a targeted pathogen, suggests increased cytotoxic and pro-atherogenic potential of MPO in diabetic females. Our analysis identified T2DM as the primary independent correlate of both MPO activity and MPO-specific activity, the latter reflecting enzyme function independent of protein concentration. These findings reinforce the view that T2DM is etiologically heterogeneous [42,43] and suggest that inflammation may be a key driver in a distinct subset of individuals. Notably, the association between MPO and T2DM remained robust, even after adjustment for factors known to influence circulating MPO levels, including regional and total adiposity measures. ROC curve analyses indicated that MPO activity and MPO-specific activity have good, though not outstanding, diagnostic performance. This is not unexpected, as MPO may be better suited as a prognostic marker, potentially predicting future inflammation-related complications, rather than as a diagnostic tool for a complex and heterogeneous disease such as T2DM.

Placing our findings within the context of the existing literature is challenging due to differences in the composition of the enrolled population (the majority of prior studies included both men and females), sample size, and the use of surrogate markers for MPO. Notably, the absence of differences in MPO concentration between diabetic and control groups has been reported in one of the largest studies conducted to date (n = 238) [24], as well as in several smaller investigations [26,44,45]. Conversely, Peng et al. observed elevated MPO levels in serum, both in free form and within extracellular vesicles [46]. Similarly. Wiersma et al. reported a modest (11%) increase in serum MPO among diabetics compared to non-diabetics [47]. However, it is worth noting that half of the T2DM participants in that study had a history of cardiovascular disease (CVD), particularly, coronary artery disease (CAD), a condition in which elevated MPO is a recognized risk factor and predictor of future cardiovascular events [48,49]. MPO is believed to actively contribute to the development and progression of atherosclerosis through mechanisms involving LDL and HDL oxidation, as well as the depletion of nitric oxide, a key molecule in maintaining endothelial function [50,51]. Therefore, increased MPO levels observed in some T2DM cohorts may reflect subclinical or undiagnosed CVD, with this potentially representing one of the source of discrepancy between studies. In support of this, Song et al. found that among T2DM patients, those with CAD had significantly higher MPO levels than those without (n = 135) [52]. Consistently, another study showed that MPO concentration was higher in TD2M patients with coronary plaque progression compared to those without this complication [53].

To date, studies specifically assessing MPO enzymatic activity remain limited, and to our knowledge, none have investigated its specific activity. Among the few relevant contributions, the study by Uchimura et al. reported elevated MPO activity in association with T2DM [54]. This increase in activity may be attributed to the stimulatory effects of hyperglycemia on hydrogen peroxide (H_2_O_2_) production [55]. Indeed, in vitro experiments have clearly shown that high glucose levels can enhance insulin-stimulated H_2_O_2_ generation in adipocytes [55]. Given that H_2_O_2_ serves as a physiological substrate for MPO, this mechanism likely contributes to the observed elevation in MPO enzymatic activity [56].

Investigating the relationship between obesity, body fat distribution, lipid profile, and myeloperoxidase (MPO) levels in females is particularly compelling, as females are uniquely susceptible to a shift from a metabolically protective gynoid fat distribution (characterized by preferential gluteo-femoral adiposity) to a dysmetabolic and pro-inflammatory android pattern (marked by central/trunk adiposity) [7,57]. This menopause-driven transition may significantly influence MPO-related inflammatory pathways. Indeed, obesity has been widely identified as a key factor influencing MPO expression [24,58,59,60]. This association is largely attributed to the fact that fat accumulation, particularly in the visceral-central compartment, frequently coincides with heightened infiltration of macrophages and neutrophils. Since MPO is the most abundant protein in neutrophils, its expression is significantly elevated in this context. Notably, we found, for the first time, that only the adiposity index significantly associated with both MPO activity and MPO-specific activity, independent of T2DM status, was the android-to-gynoid fat ratio, which reflects the relative distribution of upper versus lower body fat.

LDL-C serum concentration was the other independent predictor of MPO activity and MPO-specific activity. This rather unexpected finding, although a negative correlation with cholesterol parameters has previously been reported [44,58], remains difficult to interpret. One possible explanation is that the pro-oxidant activity of MPO, which can affect all classes of lipoproteins, may contribute to a broader disruption of cholesterol metabolism.

The present study presents some important limitations. First, its cross-sectional design limits our ability to draw definitive conclusions about the causal relationship between increased MPO activity and MPO-specific activity and the presence of T2DM. To address this, future research should adopt a longitudinal approach, ideally tracking individuals from before disease onset to better clarify temporal and causal links. Second, the relatively small sample size represents another limitation. Including a larger cohort of T2DM patients would improve the reliability and generalizability of the findings. However, the assessment of statistical power for the comparisons of MPO activity and MPO-specific activity demonstrated adequate power to detect the observed group differences. Specifically, when using a two-tailed two-sample test with α = 0.05 and estimated Cohen’s d values of 0.73 for MPO activity and 0.69 for MPO-specific activity, the corresponding statistical power was approximately 97% and 95%, respectively, indicating sufficient sensitivity to detect the observed differences between groups. Third, as the study population consisted exclusively of females, the generalizability of our findings to men remains uncertain. It cannot rule out that sex-specific differences in fat distribution, hormonal regulation, and inflammatory responses may influence MPO activity. Future studies including both men and females are warranted to determine whether these associations hold true in male populations. Moreover, the findings could be further strengthened and generalized by examining sufficiently large subgroups of controls and cases matched for age, menopausal status, and comorbidities. Fourth, information on the hypoglycemic therapies used by patients with T2DM was incomplete (see Supplementary Table S2). This limitation may affect the reliability of our findings, as several antidiabetic drugs exert pleiotropic inflammatory and metabolic effects that could influence biomarkers such as MPO [61]. However, preliminary statistical analyses assessing the potential impact of antidiabetic treatments on the observed results did not reveal any significant effects.

## 5. Conclusions

In conclusion, to the best of our knowledge, this pilot study is the first to demonstrate that MPO enzymatic activity, rather than MPO protein concentration, is independently associated with T2DM. Notably, this association persists after accounting for measures of overall and central adiposity in females, even though the android (upper) over gynoid (lower) fat distribution revealed to be an independent predictor of MPO activity.

These findings underscore the critical importance of assessing MPO functional activity, rather than mere protein levels, when investigating its role in metabolic disorders.

## Figures and Tables

**Table 1:** Population characteristics.

	Controls (n = 141)	T2DM (n = 53)
Age (years)	52 ± 11	56 ± 12 **
Menopausal status, n (%)	69 (49)	39 (74) **
Years since menopause	8 (5–11)	10 (4–13)
BMI, kg/m^2^	25 ± 4	34 ± 7 ***
Obesity, n (%)	18 (13)	37 (70) ***
Hypertension, n (%)	23 (16)	34 (65) ***
**Clinical Chemistry Indicators**		
Total cholesterol, mg/dL	232 ± 32	215 ± 54 *
HDL-C, mg/dL	67 ± 13	55 ± 13 ***
Triglycerides, mg/dL	75 (59–1001)	127 (92–190) ***
LDL-C, mg/dL	148 ± 32	132 ± 48 *
ApoA, mg/dL	175 (158–201)	151 (130–178) ***
ApoB, mg/dL	93 ± 19	98 ± 31
Glucose, mg/dL	94 (89–99)	126 (109–142) ***
HOMA-IR index	1.1 (0.7–1.7)	4.0 (2.5–7.0) ***

BMI, body mass index; LDL-C, cholesterol low-density lipoprotein; HDL-C, cholesterol high-density lipoprotein. * *p* < 0.05; ** *p* < 0.01; *** *p* < 0.001.

**Table 2:** Simple correlation coefficients (r) for the relationship between MPO activity, concentration, and -specific activity and metabolic/body fat distribution parameters in the whole sample (n = 194).

Variables	MPO Activity	MPO Concentration	MPO-Specific Activity
**Metabolic biomarkers**			
Total Cholesterol, mg/dL	−0.239 **	−0.043	−0.230 **
C-HDL, mg/dL	−0.166 *	0.033	−0.232 **
Triglycerides, mg/dL	0.076	−0.137	0.100
C-LDL, mg/dL	−0.237 **	−0.055	−0.260 **
ApoA, mg/dL	−0.203 *	−0.045	−0.194 *
ApoB, mg/dL	−0.148	−0.075	−0.076
Glucose, mg/dL	0.111	−0.137	0.176 *
Insuline	0.260 ***	−0.042	0.287 ***
HOMA-IR Index	0.257 ***	−0.055	0.270 ***
**Measures of body mass distribution**			
BMI (Kg/mq)	0.305 ***	−0.057	0.293 ***
Body Fat Mass (Kg)	0.164 *	−0.046	0.226 **
Body Fat Mass (%)	0.104	−0.100	0.204 **
Arms Fat Mass (Kg)	0.179 *	−0.046	0.241 **
Arms Fat Mass (%)	0.040	0.027	0.056
Legs Fat Mass (Kg)	0.026	−0.021	0.057
Legs Fat Mass (%)	−0.171 *	−0.021	−0.303 ***
Trunk fat mass (Kg)	0.181 *	0.079	0.260 **
Trunk fat mass (%)	0.167 *	−0.085	0.291 ***
Visceral Adipose Tissue Mass (Kg)	0.181 *	−0.086	0.277 ***
Android/Gynoid Ratio	0.236 *	−0.046	0.340 ***

BMI = body mass index. * *p* < 0.05; ** *p* < 0.01; *** *p* < 0.001.

**Table 3:** Independent predictors of MPO-specific activity in the whole sample assessed by stepwise multiple regression analysis.

Model Number	Explanatory Variables	Unstandardized Coefficients	Standard Error	Standardized Coefficients (β)	Adjusted R^2^
1	Diabetes Android/gynoid ratio LDL-C	0.244 0.416 −0.002	0.069 0.160 0.0001	0.281 ** 0.214 * −0.177 *	0.216 ***
2	Diabetes LDL-C	0.359 0.001	0.062 0.0002	0.423 *** −0.156 *	0.192 ***
3	Diabetes LDL-C	0.359 0.001	0.062 0.0002	0.385 *** −0.167 *	0.192 ***

Model 1 includes age, BMI, HOMA index, LDL-C, HDL-C, android/gynoid ratio, and diabetes. Model 2 includes age, HOMA index, LDL-C, HDL-C, visceral fat mass, legs fat percentage, and diabetes. Model 3 includes age, HOMA index, LDL-C, HDL-C, trunk fat mass, legs fat percentage, and diabetes. Criteria for variable inclusion were entry if *p* ≤ 0.05 and exclusion if *p* > 0.1. BMI = body mass index; LDL-C, cholesterol low-density lipoprotein; HDL-C, cholesterol high-density lipoprotein. * *p* < 0.05 ** *p* < 0.01; *** *p* < 0.001.

## Data Availability

The original contributions presented in this study are included in the article/Supplementary Materials. Further inquiries can be directed to the corresponding author(s).

## Supplementary Materials

The following supporting information can be downloaded at: https://www.mdpi.com/article/10.3390/antiox15010130/s1, Table S1: Body mass composition and body fat distribution in controls and T2DM patients. Table S2: Main glucose-lowering therapy in patients with T2DM (prevalence). Figure S1: Receiver operating characteristic (ROC) curves for MPO activity (left plot) and MPO-specific activity (right plot) for the diagnosis of T2DM.
