# Supplementary material for: Myeloperoxidase (MPO) Enzymatic Activity, but Not Its Protein Concentration, Is Associated with the Risk of Type 2 Diabetes in Females, Regardless of Obesity Status
Source: Antioxidants (Basel). 2026 Jan 19;15(1):130. doi: 10.3390/antiox15010130 (PMC12837212; doi:10.3390/antiox15010130)
Supplement: Supplementary file 1 [file antioxidants-15-00130-s001.zip › antioxidants-4054665-supplementary.pdf]

Supplementary Table S1. Body mass composition and body fat distribution in Controls and T2DM

	**CONTROLS** (n=141)	**T2DM** (n=53)
**Body mass composition**		
Bone Mineral Content, Kg	2.0	2.1
Bone Mineral Content (%)	3.1	2.6
Body Lean Mass (Kg)	13.8 (13.0-14.9)	16.7 (15.4-17.8)
Body Lean Mass (%)	56.4	52.9
Body Fat Mass (Kg)	26.0 (21.7-31.7)	35.1 (31.2-41.5)
Body Fat Mass (%)	40.3	44.3
	**Body fat distribution**	
Arms Fat Mass (Kg)	4.9 (4.3-6.2)	7.7 (6.2-9.0)
Arms Fat Mass (%)	20.3	21.8
Legs Fat Mass (Kg)	9.9 (8.5-11.9)	9.5 (8.9-12.1)
Legs Fat Mass (%)	38.7	29.9
Trunk fat mass (Kg)	11.3 (9.3-14.7)	17.8 (16.2-22.3)
Trunk fat mass (%)	44.2	51.5
Visceral adipose tissue mass (g)	477.0 (327.0-697.7)	1030.0 (780-1242)***
Visceral adipose tissue volume (cm^3)	516.0 (353.0-754.0)	1113 (843-.1343)***
Visceral adipose tissue area (cm^2)	99 (68-145)	214 (162-258)***
Android/gynoid ratio	0.8	1.0

*p<0.05; **p<0.01; ***p<0.001

Supplementary Table S2. Main glucose-lowering therapy in patients with T2DM (prevalence)

	T2DM (n=38)
Metformin, %	76%
Insulin, %	37%
Sulfonylureas, %	17%
SGLT2	17%
I-DPP	8%
GLP1	25%

Abbreviations: SGLT2: Sodium–glucose cotransporter 2 inhibitors; I-DPP: Dipeptidyl peptidase-4 inhibitors; GLP-1: Glucagon-like peptide-1 receptor agonists

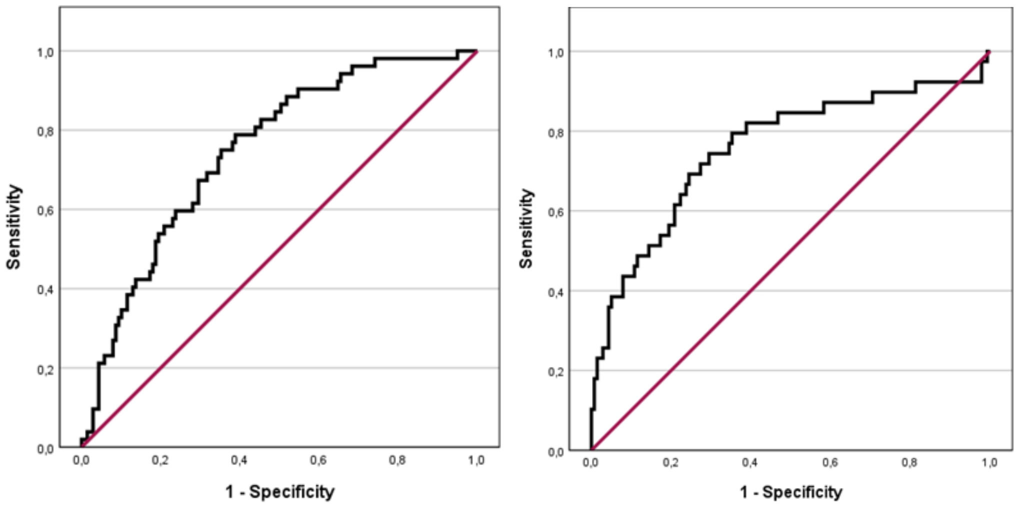

Supplementary Figure S1
Receiver operating characteristic (ROC) curves for MPO-activity (Left plot) and MPO-specific activity (Right plot) for the diagnosis of T2DM.
